# Optimization of Ceramic Paste Composition for 3D Printing via Robocasting

**DOI:** 10.3390/ma17184560

**Published:** 2024-09-17

**Authors:** Szymon Przybyła, Maciej Kwiatkowski, Michał Kwiatkowski, Marek Hebda

**Affiliations:** 1 Faculty of Materials Engineering and Physics, Cracow University of Technology, Warszawska 24, 31-155 Kraków, Poland; s.przybyla@createc.com.pl (S.P.); maciej.kwiatkowski@createc.com.pl (M.K.); 2 Createc Sp. z o.o., E. Kwiatkowskiego 9, 37-450 Stalowa Wola, Poland; m.kwiatkowski@createc.com.pl

**Keywords:** 3D printing, robocasting, oxide ceramics, viscosity

## Abstract

This article presents a procedure for selecting optimal ceramic paste formulations dedicated to the 3D printing process using robocasting technology. This study investigated pastes with varying ceramic powder particle sizes and different proportions of additives, such as ceramic microspheres and nutshells. This selection process allowed for the classification of ceramic mixtures into those suitable and unsuitable for this additive manufacturing technique. Subsequently, the viscosity of the pastes was measured, and extrudability tests were performed to determine the force required for extrusion and evaluate the quality of the extruded material. In the final stage, the setting time of the ceramic pastes was assessed to establish the drying time of the printed elements. It was found that the length of the extruded band of ceramic paste was inversely proportional to the Al₂O₃ content. Moreover, the extrusion force for samples with varying ceramic powder particle sizes (MG1–MG5) ranged from 133 to 166 N, compared to 77 N for the base sample (BM1). The obtained results enable further development in robocasting additive technology, including the development of a rapid and effective method for validating ceramic pastes used in this process.

## 1. Introduction

The continuous advancement in technology, along with increasing quality and durability demands for manufactured components, compels the industry to rapidly adapt and integrate new materials and manufacturing techniques. It is important to note that sustainable progress is only feasible when economic viability is maintained [1]. One of the modern technologies gaining prominence for the production of complex structures, which is unattainable by conventional manufacturing methods, is 3D printing [2]. In recent years, additive manufacturing (AM) technologies have undergone rapid development, with several distinct methods currently in use, including FDM (fused deposition modeling) [3], UV LCD (ultraviolet liquid crystal display) [4], SLA (stereolithography) [5], MJP (multi-jet printing) [6], binder jetting [7,8], SLS (selective laser sintering) [9], MJF (multi-jet fusion) [10], EBM (electron beam melting) [11], robocasting [12], DIW (direct ink writing) [13], and bioprinting [14]. The adoption of additive manufacturing techniques has unlocked unprecedented potential for producing parts with unique material properties, strengths, aesthetics, and design complexity [15].

The diversity of additive technologies enables the use of a wide range of materials [16]. While polymer materials [17] and metal powders [18,19] are the most common in 3D printing, oxide ceramics [20,21] are less frequently used due to the challenges associated with developing suitable processing technologies for raw ceramic powders. Utilizing ceramic materials with the most popular technique, FDM, poses significant difficulties, including the need for printer modifications, high filament costs, and suboptimal print quality. Other widely used methods where ceramic materials are applicable include SLA and binder jetting. While SLA provides high print quality, it is time-consuming and requires expensive ceramic-based resins. Binder jetting can accommodate ceramic powders that meet key criteria, such as particle size, flowability, and sinterability [21], but its large-scale production is hindered by the high costs of equipment and lengthy printing times. Robocasting has emerged as an AM technology that enables the fabrication of advanced ceramics [22] while achieving an optimal balance of cost, accuracy, and print speed. This process involves extruding a ceramic slurry (paste) from a printer cartridge and layering it sequentially, similar to FDM, but using ceramic paste instead of molten polymer.

Among ceramic materials, aluminum oxide (Al_2_O_3_), or corundum, is widely used across various industries due to its high melting point, hardness, strength, chemical stability, and wear resistance [23]. Studies have also explored the use of Al_2_O_3_ powder in the development of ceramic pastes for 3D printing applications [24,25]. Typically, ceramic powders are suspended in organic substances or polymers to pre-crosslink the paste structure, enabling the additive process. To further enhance the material properties, research is being conducted on modifying paste compositions by incorporating pore-forming additives such as ceramic microspheres [26,27] and nutshells [28] or by employing conventional foaming methods, including the blowing agent method, polymer matrix replication, the sol–gel method, foam–gel casting, and freeze-casting [29]. Introducing pores into the ceramic structure imparts unique properties, such as low density and mass, low thermal conductivity, high corrosion and wear resistance, and enhanced thermal stability and strength [29,30]. Previous studies have primarily focused on the general use of ceramic powders in additive manufacturing or specific methods for improving material properties, but they have not addressed a systematic approach to optimizing ceramic paste formulations for robocasting technology.

This article presents a procedure for selecting optimal ceramic paste formulations based on Al_2_O_3_ powder with varying particle sizes and proportions of additives, including ceramic microspheres and nutshells, tailored for 3D printing via robocasting technology. The results offer valuable insights into the development of a rapid and efficient method for validating ceramic pastes used in robocasting applications.

## 2. Materials and Methods

### 2.1. Sample Preparation

Nineteen ceramic paste variants were prepared using the following components: Al_2_O_3_ powder (CT3000 SG, Almatis, Ludwigshafen, Germany), methylcellulose (E460, ITC, Piastów, Poland), dispersant (Darvan 7ns, Vanderbilt Minerals LLC, Norwalk, Connecticut), deionized water, ceramic microspheres (E-spheres, Envirospheres Pty Ltd., Lindfield, Australia), and walnut shells (Brenntag, Essen, Germany). The samples were divided into four groups, differing in the type and quantity of ingredients used. The base chemical composition, labeled as BM1 (basic material), consisted of 78.50% Al_2_O_3_, 21.16% distilled water, 0.09% dispersant (Darvan 7-ns), and 0.25% binder (methylcellulose). The composition of BM1 was established through preliminary trials and experiments using a high-speed planetary mixer, specifically designed to produce ceramic paste based on Al_2_O_3_.

Building upon this formulation, the BM series was developed by incrementally increasing the Al_2_O_3_ content by 0.5% while maintaining the proportions of the other components (Table 1). This densification was intended to evaluate the impact of increasing Al_2_O_3_ content on the rheological properties of the ceramic paste. Notably, the BM samples did not include any additional components, such as ceramic microspheres or walnut shells. Furthermore, the Al_2_O_3_ powder used for the BM samples was utilized in its as-received state, meaning its particle size distribution (D50 = 0.4 µm) was consistent with the manufacturer’s material safety data sheet, without any further grinding to break down agglomerates formed during storage and transport.

The MG (material gradation) series was based on the same composition as BM1 but utilized Al_2_O_3_ powders with different particle size distributions, achieved by sieving the as-received powder using a laboratory shaker (LPzE-3e) to separate distinct particle size fractions (Table 2). This variation was intended to assess how particle size gradation affects the characteristics of the ceramic paste.

Samples labeled WS (walnut shell) and CM (ceramic microsphere) were modifications of the BM1 formulation, with the addition of walnut shells and ceramic microspheres at 20% and 40% volumes relative to Al_2_O_3_, respectively (Table 3 and Table 4). These additives were introduced to generate closed pores within the structure, aiming to influence both the density and mechanical properties of the printed elements.

Ceramic pastes were prepared using a Thinky ARV-310 planetary mixer (Thinky Corp., Tokyo, Japan), following a three-stage mixing process designed to ensure thorough homogenization of all ingredients. The first stage involved mixing at 750 rpm for 10 s. In the second stage, the rotational speed was increased to 1100 rpm for 30 s. The final stage lasted 60 s at 2000 rpm. The entire process was conducted automatically, without the need to remove the container or implement any pauses between stages. Upon completion, the ceramic pastes were subjected to further analysis, with any remaining material stored in vacuum-sealed bags to preserve its properties.

### 2.2. Assessment of Extrudability of Ceramic Pastes

**Figure 1:**
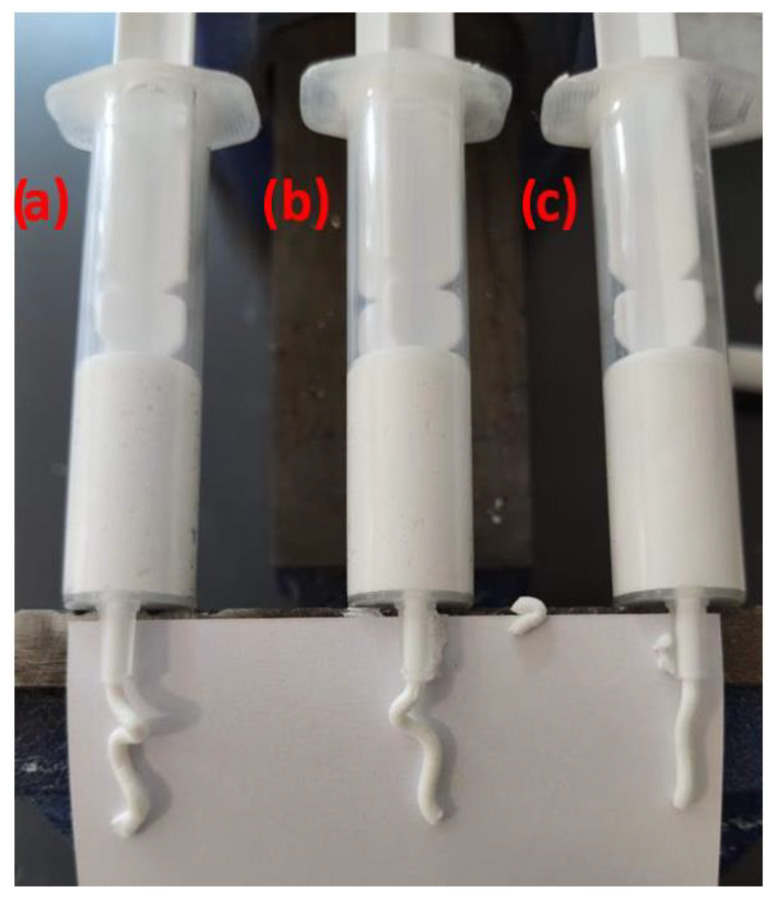
A representative view of the extrudability test of ceramic pastes depending on their composition for paste with (**a**) very low flowability (BM6), (**b**) low flowability (BM5), and (**c**) appropriate flowability (BM4).

The initial qualification phase aimed to eliminate samples that did not meet the basic requirement of being extrudable through a 2.2 mm diameter nozzle. Additionally, the consistency of the ceramic pastes was visually assessed during the measurements, along with their anticipated behavior during the printing process. Figure 1 presents a representative view of the test, illustrating three samples categorized by their flowability: (a) very low flowability, (b) low flowability, and (c) appropriate flowability. Each ceramic paste composition was extruded using a syringe filled with 5 mL of material. A critical aspect of the syringe-filling process involved the removal of air bubbles. To ensure this, the syringes were periodically vented by extruding material until all air bubbles were expelled. Once prepared, the syringes were mounted in a holder and manually compressed until 1 mL of ceramic paste was extruded.

### 2.3. Rheological Studies

To assess the flow and deformation characteristics of the ceramic pastes, a rheological analysis was performed using an Anton Paar MCR 302e rheometer with an oscillatory strain sweep test. The measurements were conducted in a PP25 plate–plate configuration, with a constant gap of 1.5 mm, a frequency of 1.6 Hz, and a temperature of 20 °C. The strain (γ) ranged from 0.01% to 100% during the study.

### 2.4. Extrusion Force Measurement

**Figure 2:**
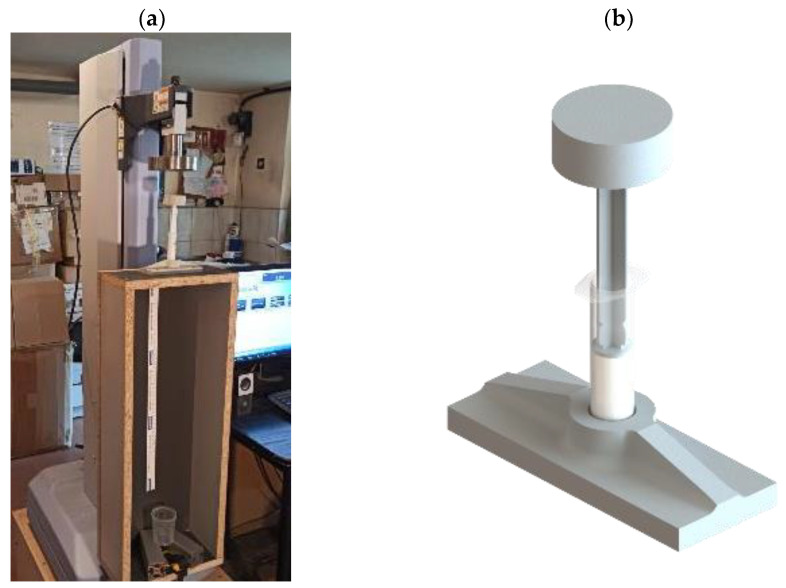
Measurement setup for extrusion force and strand length analysis: (**a**) Shimadzu EZ-LX 5 kN strength testing machine; (**b**) 3D-printed mount.

The extrusion force was measured using a Shimadzu EZ-LX (Shimadzu Corp., Kyoto, Japan) strength testing machine with a 5 kN capacity (Figure 2a). A specially designed 3D-printed holder was employed to ensure proper alignment of the syringe containing the sample (Figure 2b). The force was recorded as a function of the syringe plunger displacement, with the traverse speed set at 20 mm/min and a travel distance of 15 mm. For all samples, the initial plunger position was standardized at 35 mm from the syringe hub. Additionally, during the extrusion test, the length of the extruded ceramic paste strand was measured for each sample volume. Upon completion of the extrusion process, the plunger rebound was recorded, allowing for the assessment of paste expansion within the syringe.

### 2.5. Initial Setting Time

The initial setting time of the ceramic paste, as influenced by its composition, was determined using a Vicat apparatus. A load of 300 g was applied, and a needle with a diameter of 1.13 mm was used. Measurements were recorded at 15 min intervals, with each reading taken 30 s after the release of the locking mechanism. The tests were conducted at a controlled temperature of 20 °C. Results were based on the average of two measurements for each material variant.

## 3. Results

### 3.1. Initial Qualification

Based on the conducted tests, two primary criteria were established to qualify ceramic paste samples for subsequent material studies in the 3D printing process. The first criterion was the paste’s ability to be extruded through the nozzle without obstruction. The second criterion was the consistency, or flowability, of the ceramic paste. The results of these measurements were summarized and are presented in Table 5.

Based on the tests, only the samples labeled BM and MG were deemed suitable for further material studies. These samples demonstrated proper extrusion and maintained acceptable consistency levels. Among the BM samples, BM3 and BM4 exhibited the most favorable extrusion-to-consistency ratios. All MG samples showed slightly higher flowability compared to the BM samples, with MG4 displaying the most optimal properties.

On the other hand, the WS and CM samples were not qualified for further study. The WS samples, in particular, failed to facilitate smooth extrusion; only WS3 could be extruded through the syringe nozzle, but its consistency was excessively fluid, which would hinder the 3D printing process. The CM samples, while extrudable, also had consistency issues similar to WS3, rendering them unsuitable for 3D printing applications.

### 3.2. Rheological Studies

**Figure 3:**
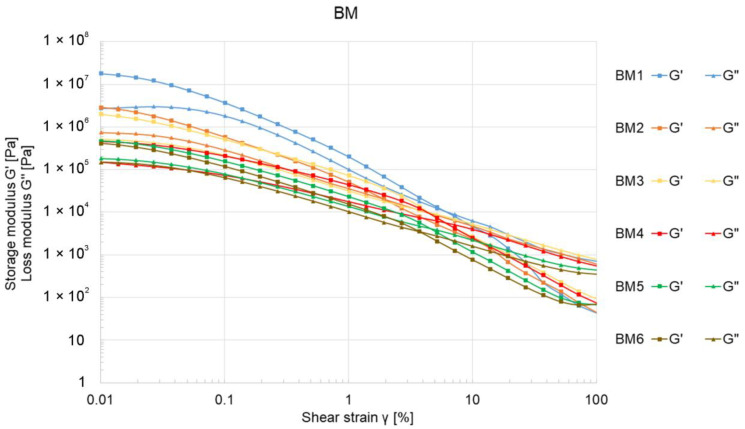
The relationship between the storage modulus G″ and the loss modulus G” as a function of shear strain γ for ceramic BM samples.

Figure 3 presents the results of the oscillatory strain sweep test, also known as the amplitude sweep, for ceramic pastes labeled BM.

**Figure 4:**
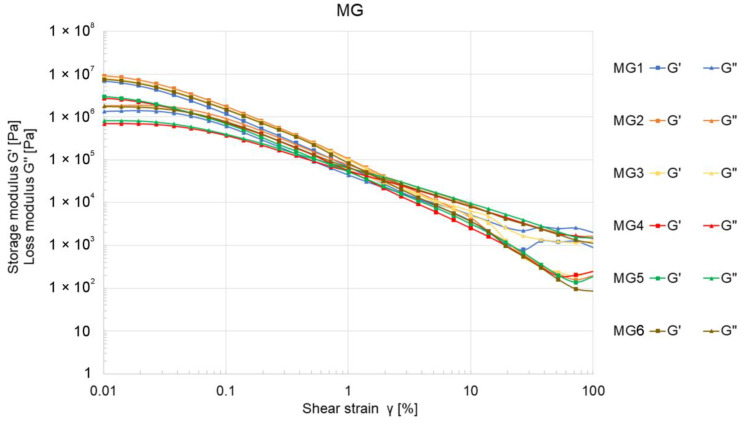
The relationship between the storage modulus G″ and the loss modulus G” as a function of shear strain γ for ceramic MG samples.

All analyzed samples exhibited viscoelastic behavior, with the storage modulus (G″) initially higher than the loss modulus (G”). As the Al_2_O_3_ content in the ceramic mixture increased, both G″ and G” decreased. Among the BM samples, BM1 displayed the highest initial modulus values, with G″ and G” recorded at 2.03 × 10^7^ Pa and 2.74∙10^6^ Pa, respectively. The intersection point of the G″ and G” curves for the BM samples was observed in the strain range of 3 to 7%. The curves for ceramic pastes labeled MG are shown in Figure 4.

All samples from the MG group also demonstrated viscoelastic behavior. Within the MG group, the values of the moduli G″ and G” were more closely matched compared to the BM samples. Among the MG samples, MG3, MG2, and MG1 exhibited higher values for both storage and loss moduli compared to other MG samples, although these values were still lower than those observed for BM1. The intersection point of the G″ and G” curves for the MG samples was found within the strain (γ) range of 0.5 to 4%.

### 3.3. Extrusion Force Measurement

The results of the extrusion force measurements for ceramic pastes, as a function of sample composition, are presented in Table 6.

**Figure 5:**
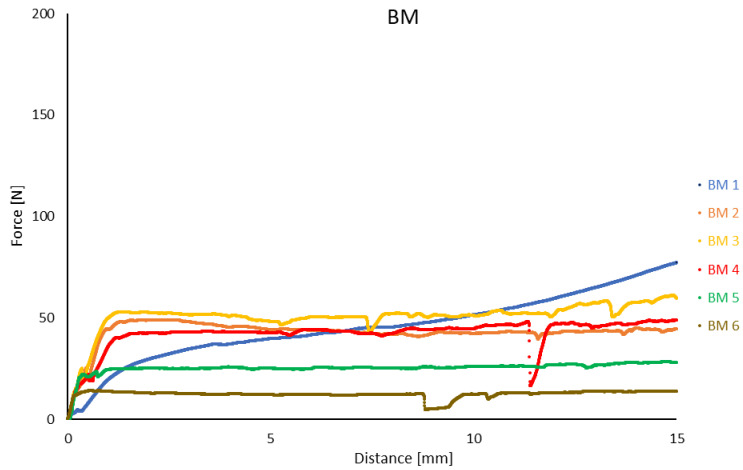
The relationship between the extrusion force of the ceramic paste and the displacement of the syringe plunger for the BM samples.

For the BM samples, the extrusion force of the ceramic paste varied between 14 N for BM6 and 77 N for BM1. It was noted that as the extrusion force increased, the length of the extruded strand also increased, ranging from 80 mm for BM6 to 365 mm for BM1. After the extrusion force measurement, the expansion of the ceramic paste within the syringe was 5 mm, which corresponded to 33% of the traverse displacement during the measurement. This effect was consistent across all tested ceramic paste compositions. The extrusion force versus displacement curves for the ceramic pastes are shown in Figure 5.

Analysis of the recorded results revealed that the extrusion force curve for the BM1 sample was unique in exhibiting a continuous increase throughout the entire test range. In contrast, for all other samples, irrespective of their composition, the extrusion force stabilized after approximately 1 to 1.5 mm of traverse displacement. Within this range, the maximum extrusion force was recorded for the BM2 and BM6 samples. Beyond this point, the extrusion force for these samples exhibited a slight decrease. For the remaining samples, the maximum extrusion force occurred at the final measurement point.

**Figure 6:**
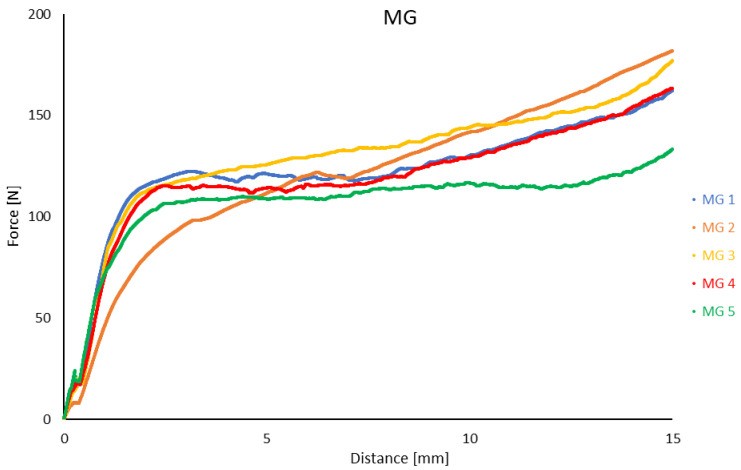
The relationship between the extrusion force of ceramic paste and the displacement of the syringe plunger for samples labeled as MG.

The extrusion force curves for all MG samples exhibited a continuous increase throughout the entire test range, similar to the behavior observed for the BM1 sample, as depicted in Figure 6.

The MG samples generally exhibited higher extrusion forces compared to the BM samples. The lowest extrusion force was recorded for the MG5 sample, at 133 N, while the highest was observed for the MG3 sample, at 177 N. For the MG1, MG2, and MG3 samples, the strand length exceeded the maximum measurable value on the scale and was recorded as over 520 mm. The strand lengths for the MG4 and MG5 samples were 340 mm and 370 mm, respectively (Table 6). The expansion of the ceramic paste within the syringe for all MG samples was consistently 5 mm.

### 3.4. Initial Setting Time

The initial setting time was measured for three ceramic paste samples that exhibited the best technological properties during preliminary qualification (BM3, BM4, and MG4). The average initial setting times were comparable across the samples, recorded as 33 h and 45 min for BM3, 33 h and 15 min for BM4, and 34 h and 30 min for MG4. In each instance, the distance between the needle and the base plate at the conclusion of the test was 9 mm.

## 4. Discussion

### 4.1. General

Selecting the appropriate chemical composition of ceramic paste is critical in manufacturing components using robocasting technology. The paste must possess a consistency that is sufficiently thick to support the formation of layers while maintaining the intended dimensions and geometry. At the same time, it should be fluid enough to facilitate extrusion with minimal force. This optimal consistency allows for higher printing speeds and the use of larger material reservoirs and smaller diameter nozzles, leading to increased economic efficiency, enhanced resolution of printed elements, improved surface quality, and the capability to print more complex geometries.

Additionally, proper adjustment of the retraction parameter is essential in robocasting technology. This parameter significantly affects the shape and dimensions of printed elements at the end of the material extrusion, influencing the amount of ceramic paste transferred beyond the extruder due to uncontrolled flow from the printing nozzle. To improve the quality of printed elements and streamline the process, it is crucial to minimize material expansion within the printer cartridge by carefully selecting the ceramic paste composition. The results presented demonstrate that both the proportions of ingredients and the particle size of the ceramic powder impact rheological parameters, extrusion force, and initial setting time.

### 4.2. Rheological Parameters

Ceramic pastes of the BM grade exhibited varying degrees of fluidity. Samples BM1 and BM2, despite their higher fluidity, were deemed suitable for 3D printing applications. Conversely, pastes with higher Al_2_O_3_ content (BM5 and BM6) exhibited lower fluidity. The most optimal consistency was observed in samples BM3 and BM4. For the MG grade, all mixtures showed increased fluidity compared to BM1. Among the MG grades, sample MG3 demonstrated the most favorable fluidity for robocasting technology.

Both BM and MG ceramic pastes initially displayed viscoelastic behavior in rheological tests. However, the recorded curves in Figure 3 and Figure 4 lacked a characteristic linear viscoelastic region (LVE), suggesting weak internal bonding forces within the ceramic pastes. This could be attributed to the mixing methodology and the composition of the mixtures. The mixing process was conducted using a planetary mixer, which completed the process quickly while simultaneously degassing the mixture. Following mixing, the samples were promptly transferred to containers and sealed in vacuum bags. This minimal exposure to air and oxygen—an activator in the setting process when using methylcellulose as a binder—may have contributed to the absence of a distinct LVE region.

Additionally, a decrease in G″ and G” moduli (Figure 3) was observed to be directly proportional to the increase in the percentage of ceramic powder in the mixture (Table 1). These results suggest that an inadequate proportion of ceramic powder to other ingredients, particularly the binder, weakens the bonding forces within the mixture, making it more susceptible to deformation. This effect could adversely affect the formation and propagation of cracks during the drying and firing stages of the printed elements. A similar weakening of bonding forces was noted with larger ceramic powder gradations (samples MG4 and MG5), whereas smaller Al_2_O_3_ powder particles positively influenced the increase in G″ and G” moduli (Figure 4). The extrusion force measurements for BM-labeled samples did not reveal a significant correlation between extrusion force and Al_2_O_3_ content in the pastes, with values not exceeding 77 N (Figure 5).

### 4.3. Extrusion Parameters

Analysis of the recorded results revealed that the length of the extruded paste strand decreased inversely with increasing Al_2_O_3_ content in the mixture (Table 6). Additionally, the behavior of the extrusion force as a function of syringe plunger displacement for the BM1 sample was notably different. For BM1, a continuous increase in force was observed, whereas other mixtures in the same group showed a 20% increase in force after the stabilization period (Figure 5). In contrast, extrusion force values for MG samples were significantly higher than those for BM1, ranging from 133 to 177 N (Table 6), representing an increase of 1.7 to 2.3 times compared to BM1. This indicates that the particle size range of the powder has a substantial impact on extrusion force.

Moreover, all MG mixtures facilitated the production of significantly longer extruded material strands. For MG1, MG2, and MG3, the strand lengths exceeded the maximum measurable range of 520 mm, while MG4 and MG5 exhibited extrusion lengths of 340 mm and 370 mm, respectively, similar to the BM1 grade (Table 6). The length of the extruded strand is a critical parameter in robocasting technology. A non-continuous strand can result in pores and voids, which may become sites for crack initiation, warping, or other defects during drying and sintering. Conversely, excessive strand length indicates high bonding strength within the ceramic paste, which can lead to surface defects such as warping and geometry errors at the layer interfaces. Therefore, samples with excessively long strand lengths, such as BM4, BM5, MG1, MG2, and MG3 (Table 6), may not meet the requirements of the additive manufacturing process, which necessitates a continuous printed path with smooth transitions at the start and end of each layer.

For all samples, regardless of grade (BM or MG), the retraction of the syringe plunger after extrusion was 5 mm. This retraction resulted in an expansion value of over 33% relative to the plunger movement, which was 15 mm during the test. Such a high expansion value may negatively affect self-extrusion during 3D printing, leading to uncontrolled material flow. To mitigate nozzle leaks, the retraction parameter must be adjusted to compensate for material expansion during the printing process.

The initial setting times for the tested materials were comparable across compositions, with BM2 at 33 h and 45 min, BM3 at 33 h and 15 min, and MG3 at 34 h and 30 min. An advantage of 3D printing is its capability to create components with a partially internal fill structure, which not only reduces the mass and material usage but also significantly shortens the drying time of printed objects.

### 4.4. Additives for Ceramic Paste

Furthermore, the introduction of additives such as ceramic microspheres or nutshells into ceramic paste mixtures presents specific limitations concerning their applicability (Table 5). For nutshells with particle sizes ranging from 450 to 1000 µm, it is necessary to use printing nozzles with diameters greater than 2.2 mm. The addition of nutshell particles with sizes ranging from 200 to 450 µm at a volumetric ratio of 20% relative to Al_2_O_3_ (sample WS3) excessively fluidized the ceramic paste, impairing the proper formation of the extruded strand. Additionally, a ceramic paste containing 40% nutshell particles of 200 to 450 µm (sample WS4) did not meet the criteria for free extrusion (Table 5). This issue was exacerbated by the significant segregation of the nutshells within the mixing container, a problem observed in all WS samples.

To utilize nutshells as an organic filler to reduce Al_2_O_3_ content and introduce porosity in the sintered element, it is crucial to optimize the mixing intensity to prevent segregation of the paste on the walls of the planetary mixer container. For all CM samples containing ceramic microspheres as an additive, the ceramic paste exhibited excessive fluidity (Table 5), rendering it unsuitable for robocasting technology. Therefore, to effectively use ceramic microspheres for creating controlled porosity in the ceramic paste, a new paste composition must be developed to address its high fluidity.

### 4.5. Evaluating Method

This research also facilitated the evaluation of methods for assessing ceramic pastes under production conditions. An effective method should be characterized by ease of execution, minimal preparation and testing time, and no requirements for expensive or complex laboratory equipment. Among the methods evaluated, syringe extrusion emerged as the fastest and simplest for assessing the suitability of ceramic mixtures for 3D printing. However, this method necessitates individual adjustments of force values, feed speed, and the length of the extruded ceramic paste.

## 5. Conclusions

Based on the research conducted, it was found that the syringe test can be used as an initial qualification method for ceramic paste for robocasting 3D printing processes. However, its results significantly depend on the additives used in the ceramic pastes, the characteristics of the mixing process, and the parameters of the 3D printing process. Rheological tests showed that all mixtures exhibited weak internal bonds, indicating the need to modify either the mixing process or the chemical composition. This is particularly evident in the absence of the LIVE region in the amplitude sweeps diagrams. Moreover, a noticeable decrease in the storage modulus (G″) and loss modulus (G”) values was observed—from 2.03∙10^7^ Pa (G″) and 2.74∙10^6^ (G”) Pa for the sample with 78.5% Al_2_O_3_ content (BM1) to 1.57∙10^7^ Pa (G″) and 2.72∙10^7^ Pa (G”) for the sample with 81% Al_2_O_3_ content (BM6). The extrusion force of samples with the same chemical composition, but differing in ceramic powder particle size (samples MG1-MG5), was an order of magnitude higher than that of the base sample (range 133–166 N), compared to the base sample made of alumina oxide as delivered (BM1), for which the extrusion force was 77 N. Furthermore, the length of the extruded band of ceramic paste was inversely proportional to the Al_2_O_3_ content. The initial setting time was independent of the composition variant tested.

## Figures and Tables

**Table 1:** Chemical composition of ceramic paste for BM samples.

Sample Designation	Chemical Composition [%]			
	Al_2_O_3_	H_2_O	Dispersant	Methylcellulose
BM1	78.50	21.16	0.09	0.25
BM2	79.00	20.64	0.09	0.24
BM3	79.50	20.15	0.09	0.24
BM4	80.00	19.68	0.09	0.23
BM5	80.50	19.24	0.08	0.23
BM6	81.00	18.81	0.08	0.22

**Table 2:** Chemical composition of ceramic paste for MG samples.

Sample Designation	Chemical Composition [%]				Particle Size of Al_2_O_3_ [µm]
	Al_2_O_3_	H_2_O	Dispersant	Methylcellulose	
MG1	78.50	21.16	0.09	0.25	<100
MG2	78.50	21.16	0.09	0.25	100–250
MG3	78.50	21.16	0.09	0.25	250–500
MG4	78.50	21.16	0.09	0.25	500–600
MG5	78.50	21.16	0.09	0.25	600–1000

**Table 3:** Chemical composition of ceramic paste for WS samples.

Sample Designation	Chemical Composition [%]					Particle Size of Walnut Shells [µm]
	Al_2_O_3_	H_2_O	Dispersant	Methylcellulose	Walnut Shells	
WS1	65.85	24.30	0.10	0.29	9.46	450–1000
WS2	53.12	26.14	0.11	0.31	20.32	450–1000
WS3	66.65	24.60	0.10	0.29	8.36	200–450
WS4	54.51	26.82	0.11	0.32	18.24	200–450

**Table 4:** Chemical composition of ceramic paste for CM samples.

Sample Designation	Chemical Composition [%]					Particle Size of Ceramic Microspheres [µm]
	Al_2_O_3_	H_2_O	Dispersant	Methylcellulose	Ceramic Microspheres	
CM1	68.61	25.32	0.11	0.30	5.66	<75
CM2	58.14	28.61	0.12	0.34	12.79	<75
CM3	68.95	25.45	0.11	0.30	5.19	75–150
CM4	58.14	28.93	0.12	0.34	11.81	75–150

**Table 5:** Results of qualification of ceramic pastes for the robocasting 3D printing process.

Sample	Extrusion Process Evaluation	Assessment of Ceramic Paste Consistency	Final Evaluation of Ceramic Paste Qualification
BM1	OK	OK	OK
BM2	OK	OK	OK
BM3	OK	OK	OK
BM4	OK	OK	OK
BM5	OK	OK	OK
BM6	OK	OK	OK
MG1	OK	OK	OK
MG2	OK	OK	OK
MG3	OK	OK	OK
MG4	OK	OK	OK
MG5	OK	OK	OK
WS1	NOK	NOK	NOK
WS2	NOK	NOK	NOK
WS3	OK	NOK	NOK
WS4	NOK	NOK	NOK
CM1	OK	NOK	NOK
CM2	OK	NOK	NOK
CM3	OK	NOK	NOK
CM4	OK	NOK	NOK

Note: OK—sample met the requirements, NOK—sample did not meet the requirements.

**Table 6:** The results of the extrusion force measurement for ceramic pastes of types BM and MG.

Sample	F_max_ of Extrusion [N]	Extrudate Length [mm]	Extrudate Expansion	
			[mm]	[%]
BM1	77	365	5	33.33
BM2	49	325	5	33.33
BM3	61	260	5	33.33
BM4	49	270	5	33.33
BM5	28	140	5	33.33
BM6	14	80	5	33.33
MG1	162	>520	5	33.33
MG2	166	>520	5	33.33
MG3	177	>520	5	33.33
MG4	163	340	5	33.33
MG5	133	370	5	33.33

## Data Availability

The original contributions presented in the study are included in the article, further inquiries can be directed to the corresponding author.
